# Effect of mixed Mycobacterium tuberculosis infection on rapid molecular diagnostics among patients starting MDR-TB treatment in Uganda

**DOI:** 10.21203/rs.3.rs-3324330/v1

**Published:** 2023-09-28

**Authors:** Kevin Komakech, Lydia Nakiyingi, Ashab Fred, Beatrice Achan, Moses Joloba, Bruce J Kirenga, Willy Ssengooba

**Affiliations:** Makerere University; Makerere University; Makerere University; Makerere University; Makerere University; Makerere University Lung Institute, Makerere University College of Health Sciences; Makerere University

**Keywords:** Mixed MTB infection, GeneXpert, LPA, Accuracy

## Abstract

**Background:**

We evaluated the effect of mixed-MTB strain infection on the performance of Line Probe Assay (LPA) and GeneXpert MTB/RIF (Xpert) assays among patients initiating MDR-TB treatment in Uganda

**Methods:**

This was a cross-sectional study using sputum specimens collected from participants screened for STREAM 2 clinical trial between October 2017 and October 2019. Samples from 62 MTB smear-positive patients and rifampicin-resistant patients from the peripheral health facilities were processed for Xpert and LPA as screening tests for eligibility in the trial. From November 2020, processed stored sputum samples were retrieved and genotyped to determine the presence of mixed-MTB strain infection using a standard 24-locus Mycobacterial Interspersed Repetitive Unit–Variable Number Tandem-Repeat (MIRU-VNTR). Samples with at least 20/24 MIRU-VNTR loci amplified were considered for analysis. Agar proportional Drug Susceptibility Test (DST) was performed on culture isolates of samples that had discordant results between LPA and Xpert. The impact of the presence of mixed-MTB strain on Xpert and LPA test interpretation was analyzed.

**Results:**

A total of 53/62 (85%) samples had analyzable results from MIRU-VNTR. The overall prevalence of mixed-MTB infection was 5/53 (9.4%). The prevalence was highest among males 3/33 (9.7%) and among middle-aged adults, 4/30 (13.3%). Lineage 4 of MTB contributed 3/33 (9.1%) of the mixed-MTB infection prevalence. Having mixed MTB strain infection increased the odds of false susceptible Xpert test results (OR 7.556, 95% CI 0.88–64.44) but not for LPA. Being HIV-positive (*P=0.04*) independently predicted the presence of mixed MTB infection.

**Conclusions:**

The presence of mixed-MTB strain infection may affect the performance of the GeneXpert test but not for LPA. For patients with high pre-test probability of rifampicin resistance, an alternative rapid method such as LPA should be considered.

## INTRODUCTION

Tuberculosis (TB) stands out as a major cause of ill health and mortality globally [1]. It is estimated that about a quarter of the world’s population is infected with TB [2]. The situation is worsened due to the fact that its incidence rate rose by 3.6% between 2020 and 2021 causing illness in over 10.6 million people. The death toll was as well high claiming over 1.4 million lives in 2021 [2] which is thought to be due to primary infection with TB, endogenous reactivation of primary infection or exogenous re-infection with a new strain of TB [3]. According to Stop TB Partnership Uganda, about 90,000 TB cases were detected and 7,400 TB deaths were registered [4]. In addition, the World Health Organization also grouped Uganda among the 30 high burden TB and TB/HIV infected countries [2].

The global incidence of TB has greatly increased due to emergence of drug-resistant *Mycobacterium tuberculosis* (MTB) strains [5]. When a TB strain does not respond to both isoniazid (INH) and rifampicin (RIF) anti-TB drugs, it is said to be multidrug resistant (MDR). However, when a TB strain is MDR but still resistant to fluoroquinolones and any of the three injectable second-line drugs, it is said to be extensively drug-resistant [6]. This makes TB drug resistance a major threat to global tuberculosis care and control [7]. For example, about 240,000 deaths and 490,000 new cases of MDR-TB were reported in 2020 worldwide [8]. The prevalence was also high in Uganda with about 1,900 cases [9]. However, apart from drug-resistant MTB strains, infection with multiple MTB strains could also account for the global rise in the incidence of TB and the emergency of drug-resistant MTB (15).

When one is infected with more than one clonally distinct *M. tuberculosis* strain, he or she is said to have mixed MTB infection [10]. This infections can occur when more than one distinct MTB strain is transmitted through a single event or through multiple events during a single disease episode [3]. Mixed infections can also assist MTB strains to acquire additional mutations, facilitate the spread of drug-resistant strains and boost the rate of treatment failure [11, 12]. Several methods have been employed to detect mixed MTB infection though MIRU-VNTR has been the most widely used [11]. In some cases, fast-acquired drug resistance could also be caused by hetero-resistance which is the presence of both susceptible and resistant MTB strains in the same sample [13]. There are two mechanisms for hetero-resistance; that is to say, mixed infection and splitting of a single strain into susceptible and resistant clones due to evolution [14]. The presence of hetero-resistance poses a challenge in diagnosis and treatment where it’s very difficult to detect it by phenotypic DST or GeneXpert MTB/RIF assay which are commonly used in our setting [11]. However, DNA sequencing though a robust method for analyzing sequence variants with results showing dual peaks in the *rpoB* gene region, is regarded as the gold standard for detecting rifampin hetero-resistance [14].

Mixed infections and hetero-resistance may be particularly common in regions with a high rate of TB [11, 14, 15] like Uganda. Hence; a prevalence of infection with multiple MTB strains in such a population could have implications for interpretation of rapid molecular diagnostic test such as Line Probe Assay (LPA) and GeneXpert for detecting drug resistance. This is because, only one strain may be detected despite coexistence of both drug resistant and susceptible strains which in the long run, affects decisions regarding tuberculosis control and predictions for patients' clinical responses to therapy and hence increase in drug resistance [3, 16]. This study was thus aimed at determining the prevalence of mixed MTB infection and their implications for the interpretation of rapid molecular diagnostic test among patients being initiated for MDR-TB treatment at Mulago National TB treatment unit.

## MATERIALS AND METHOD

### Study site and design

This was a cross-sectional study using pellets of sputum specimens collected from participants screened for STREAM 2 clinical trial between October 2017 and October 2019. All laboratory procedures were performed at Mycobacteriology (BSL-3) Laboratory at the Department of Medical Microbiology, Makerere University, Kampala, Uganda. From November 2020, processed stored sputum samples were retrieved and genotyped to determine the presence of mixed-MTB infection using a standard 24-locus Mycobacterial Interspersed Repetitive Unit–Variable Number Tandem-Repeat (MIRU-VNTR).

### DNA extraction

Briefly, 1ml of the sputum pellet was re-suspended into 200μl of Tris-EDTA buffer (10 mM Tris-HCl, 1 mM EDTA, pH 7.0) in a conical Eppendorf tube. The cell suspension was centrifuged at 13200rpm for 5 minutes before re-suspending the pellet into 200μl of Tris-EDTA (pH 7.0). The suspended pellet was incubated at 95°C for a minimum of 1 hour using a Hybridization oven before re-centrifuging at 13000 rpm for 1 minute to pellet the cell debris. Finally, the supernatant containing the DNA was harvested and transfer into a sterile tube and stored at −20°C until further use [17].

### PCR amplification

Using the Genoscreen MIRU-VNTR Typing Kit, the 24 markers were amplified from purified DNA using 6 quadruplex PCR and fluorescent primers specific for the flanking regions of the targeted loci in the Pre-Amplification room. In the Amplification room, 2.0μL of DNA extract was added to each reaction tube. However, 2.0μL of H37Rv DNA was added into the positive control tubes before loading the PCR tubes into the PCR machine after ensuring they were tightly closed. PCR products were stored at 4°C or − 20°C until analysis [18].

### PCR product analysis and PCR product sizing

PCR product analysis was done using the electrophoresis capillary sequencer. Briefly, 2 μL of PCR product were migrated with a mixture of HiDi formamide and GeneScan 1200 LIZ on ABI 3730XL capillary sequencer to analyse and determine the PCR product size respectively. The experiment was setup as follows; Capillary length − 50 cm; Oven temperature − 63°C; Injection voltage – 1.6 kV; Run voltage − 10 kV; Injection time − 25 s and Run time for 6500 s.

### Band interpretation of electrophorized products

From the capillary image peaks, the corresponding MIRU-VNTR peaks were interpreted as copy numbers based on the reference allele calling table in the Supply 2005 protocol [19]. Mixed-strain *M. tuberculosis* infections were categorized on the basis of the presence of multiple repeats at MIRU-VNTR loci which indicated genetic heterogeneity. Mixed infections were defined as the presence of ≥ 2 repeats at > 1 locus in the same sputum sample. To account for possible genetic heterogeneity due to the microevolution of a single infecting strain, the presence of > 1 repeat at a single locus was defined as a possibly mixed-strain infection. Single-strain infections were defined as those with single repeat patterns in all MIRU-VNTR loci.

### Strain Identification using the MIRU-VNTR genotypes

To determine MTB strain lineages, relatedness or clustering, the MIRU-VNTR genotypes were matched with reference strains in the MIRU-VNTR*plus* database (http://www.miru-vntrplus.org/) using a categorical coefficient of 1 and a distance cut off of < 0.3 that corresponds to a seven-locus difference [20].

### Agar Proportional Method

This is the current gold standard method for drug susceptibility testing and was used to test the resistance/susceptibility status of isolates to two first-line drugs (RIF and INH). This test was performed on Middlebrook 7H10 medium according to the Clinical and Laboratory Standards Institute (CLSI) procedures and recommended critical concentrations by World Health Organization (RIF-1.0μg/mL, INH-0.2μg/mL) [21, 22]. Bi-plates were used where drug concentrations were incorporated in the media to suit a final concentration of the respective drugs in each quadrant. An inoculum concentration of 1 McFarland was used, where 100μl of it was dispensed on the plate and homogenously streaked before incubating the plates at 37°C for 21 days. H37Rv laboratory strain was used as a positive control where it was inoculated on a drug free plate. After 21 days, any ≥ 1% growth on the drug containing plate compared to the drug free plate was interpreted as resistant and the reverse was true for a susceptible strain.

### Effect of Mixed MTB infection on the performance of Rapid molecular diagnostic tests

This was assessed by determining association between having mixed MTB infection and the rapid molecular diagnostic test results. A test result was categorized as “Affected” if it showed “Indeterminate” (the diagnostic test could not distinguish whether the sample was resistant or susceptible) or when it showed a “False Negative/Positive” result as compared to the gold standard test – Agar Proportional Method (APM). Results were presented as proportions and percentages.

## RESULTS

### Study site and design

This was a cross-sectional study using pellets of sputum specimens collected from participants screened for STREAM 2 clinical trial between October 2017 and October 2019. All laboratory procedures were performed at Mycobacteriology (BSL-3) Laboratory at the Department of Medical Microbiology, Makerere University, Kampala, Uganda. From November 2020, processed stored sputum samples were retrieved and genotyped to determine the presence of mixed-MTB infection using a standard 24-locus Mycobacterial Interspersed Repetitive Unit–Variable Number Tandem-Repeat (MIRU-VNTR).

### DNA extraction

Briefly, 1ml of the sputum pellet was re-suspended into 200μl of Tris-EDTA buffer (10 mM Tris-HCl, 1 mM EDTA, pH 7.0) in a conical Eppendorf tube. The cell suspension was centrifuged at 13200rpm for 5 minutes before re-suspending the pellet into 200μl of Tris-EDTA (pH 7.0). The suspended pellet was incubated at 95°C for a minimum of 1 hour using a Hybridization oven before re-centrifuging at 13000 rpm for 1 minute to pellet the cell debris. Finally, the supernatant containing the DNA was harvested and transfer into a sterile tube and stored at −20 °C until further use [17].

### PCR amplification

Using the Genoscreen MIRU-VNTR Typing Kit, the 24 markers were amplified from purified DNA using 6 quadruplex PCR and fluorescent primers specific for the flanking regions of the targeted loci in the Pre-Amplification room. In the Amplification room, 2.0μL of DNA extract was added to each reaction tube. However, 2.0μL of H37Rv DNA was added into the positive control tubes before loading the PCR tubes into the PCR machine after ensuring they were tightly closed. PCR products were stored at 4°C or − 20°C until analysis [18].

### PCR product analysis and PCR product sizing

PCR product analysis was done using the electrophoresis capillary sequencer. Briefly, 2 μL of PCR product were migrated with a mixture of HiDi formamide and GeneScan 1200 LIZ on ABI 3730XL capillary sequencer to analyse and determine the PCR product size respectively. The experiment was setup as follows; Capillary length - 50 cm; Oven temperature - 63°C; Injection voltage – 1.6 kV; Run voltage - 10 kV; Injection time - 25 s and Run time for 6500 s.

### Band interpretation of electrophorized products

From the capillary image peaks, the corresponding MIRU-VNTR peaks were interpreted as copy numbers based on the reference allele calling table in the Supply 2005 protocol [19]. Mixed-strain *M. tuberculosis* infections were categorized on the basis of the presence of multiple repeats at MIRU-VNTR loci which indicated genetic heterogeneity. Mixed infections were defined as the presence of ≥2 repeats at >1 locus in the same sputum sample. To account for possible genetic heterogeneity due to the microevolution of a single infecting strain, the presence of >1 repeat at a single locus was defined as a possibly mixed-strain infection. Single-strain infections were defined as those with single repeat patterns in all MIRU-VNTR loci.

### Strain Identification using the MIRU-VNTR genotypes

To determine MTB strain lineages, relatedness or clustering, the MIRU-VNTR genotypes were matched with reference strains in the MIRU-VNTR*plus* database (http://www.miru-vntrplus.org/) using a categorical coefficient of 1 and a distance cut off of < 0.3 that corresponds to a seven-locus difference [20].

### Agar Proportional Method

This is the current gold standard method for drug susceptibility testing and was used to test the resistance/susceptibility status of isolates to two first-line drugs (RIF and INH). This test was performed on Middlebrook 7H10 medium according to the Clinical and Laboratory Standards Institute (CLSI) procedures and recommended critical concentrations by World Health Organization (RIF-1.0μg/mL, INH-0.2μg/mL) [21, 22]. Bi-plates were used where drug concentrations were incorporated in the media to suit a final concentration of the respective drugs in each quadrant. An inoculum concentration of 1 McFarland was used, where 100μl of it was dispensed on the plate and homogenously streaked before incubating the plates at 37°C for 21 days. H37Rv laboratory strain was used as a positive control where it was inoculated on a drug free plate. After 21 days, any ≥1% growth on the drug containing plate compared to the drug free plate was interpreted as resistant and the reverse was true for a susceptible strain.

### Effect of Mixed MTB infection on the performance of Rapid molecular diagnostic tests

This was assessed by determining association between having mixed MTB infection and the rapid molecular diagnostic test results. A test result was categorized as “Affected” if it showed “Indeterminate” (the diagnostic test could not distinguish whether the sample was resistant or susceptible) or when it showed a “False Negative/Positive” result as compared to the gold standard test – Agar Proportional Method (APM). Results were presented as proportions and percentages.

## RESULTS

Table 1 describes the characteristics of the participants. A total of 62 isolates were genotyped of which 53 were able to be characterized. The participant’s ages range from 18 to 55 with an average age of 34 years and a majority of 56.6% (n=30) in the 31– 45 age group. Most participants were males 58.5% (n=33) and most of the strains identified were from lineage 4 (62.3%, n=33).

The overall prevalence of mixed *Mycobacterium tuberculosis* infection was 9.4% (n=5). The prevalence varied among males 9.7% (n=3) and females 9.1% (n=2) and was highest among middle-aged adults 13.3% (n=4). Lineage 4 had the highest frequency of isolates with mixed MTB infection though lineage 2 had the highest percentages (Table 2). All characteristics were not associated (P>0.05) with the occurrence of mixed MTB infection.

Using Agar Proportional Method (APM), 57% (30/53) and 43% (23/53) of the isolates were MDR-TB and rifampicin-resistant respectively. Line Probe Assay detected 47% (25/53) and 36% (19/53) of the isolates as MDR-TB and RR strains respectively. In addition, it also reported 8% (4/53), 2% (1/53), and 6% (3/53) of the isolates as isoniazid-resistant, indeterminate, and sensitive to both isoniazid & rifampicin respectively. GeneXpert reported 90% (48/53), 6% (3/53), and 4% (2/53) of the isolates as RR, indeterminate, and sensitive to both isoniazid & rifampicin respectively (Table 2).

Having mixed MTB infection increased the odds of false susceptible Xpert test results (OR 7.556, 95% CI 0.88–64.44) but not for LPA, Table 3.

Among the factors associated with MTB mixed infection among RR-TB patients, being HIV-positive (OR 24.00, *P=0.04 95% CI* 1.656–347.89) independently predicted presence of mixed MTB infection, Table 4.

## DISCUSSION

In this study among MDR/RR-TB initiated on treatment, we found that the presence of mixed-MTB infection may affect the performance of the GeneXpert test but not for LPA. Sequential infection by genetically distinct MTB strains may lead to the development of drug-resistant tuberculosis [11, 23]. This may occur if low-level drug-resistant subpopulations in clinical MTB samples are undetected and untreated [24]. The above study assessed the effect of mixed MTB infection on the performance of rapid molecular diagnostic tests.

The prevalence of mixed MTB infection was high at 9.4% compared to a prevalence of 7.1% reported by Dickman *et al* [16] in 2010 and of 4% reported by Ssengooba *et al* [25] in 2015. The difference in the prevalence could be attributed to the detection method used or the type of sample used. This is because Dickman *et al* amplified 15 MIRU-VNTR loci using a single target ordinary PCR which is inferior for the detection of mixed MTB infection compared to amplifying 24 MIRU-VNTR loci which has a higher resolution [24]. Ssengooba *et al* used blood and sputum samples from patients who were none MDR-TB hence the prevalence could be lower since [26] reported a higher prevalence of 15% among MDR-TB patients in South Africa. However, this prevalence is slightly lower compared with that of 11.1% reported by Muwonge *et al* [27] in 2014. The prevalence was highest among middle-aged adults and within Lineage 4 though none of the above variables were significantly associated with it. These findings agree with [28] who reported no association of mixed MTB infection with age and sex. However it disagrees with [29] who reported an association between mixed MTB infection and age and sex. In addition, [30] also reported strong association between having mixed MTB infection and CAS1_Delhi lineage (Lineage 3) which disagrees with this findings. The difference in these findings could be attributed to the geographical location where the study was done since the different MTB lineages are geographically located except Lineage 4 which seems to be widely distributed [31].

GeneXpert MTB/RIF and LPA were the rapid diagnostic tests whose performances were assessed in the presence of mixed MTB infection. The study showed that having mixed MTB strain infection increased the odds of affecting GeneXpert results by either giving indeterminate results or false results. This finding is in agreement with [24, 32, 33] who reported that the presence of a mixture of resistant subpopulations may create difficulties in the interpretation of rapid molecular drug resistance tests because it leads to ‘indeterminate’ test results. However, there was no significant association between having mixed MTB infection and having LPA test results affected. This could be because LPA detects resistant MTB subpopulations when they comprise ≥ 5% of the bacilli in the sample [34] unlike with GeneXpert which requires > 50% of the resistant subpopulation for its detection [11, 35–37]. Hence the presence of minority-resistant strains was most likely missed out by the GeneXpert test.

Risk factors associated with mixed MTB infection were also assessed. Overall, participants that had MDR-TB 10.0% (3/30), middle age adults (31–45) 13.3% (4/30), and those that were HIV positive 25% (4/16) had more cases of mixed MTB infection (Table 4). This findings agree with [29] who reported that mixed MTB infections were significantly more likely to occur in middle age adults than in other age groups. However, this finding disagrees with [28, 38] who reported no significant association between having HIV and mixed MTB infection. The study also showed that having MDR-TB did not predict the presence of mixed MTB infection which is in agreement with [39] who reported no association between mixed MTB infection and having MDR-TB. Testing sensitive for rifampicin resistance by GeneXpert given you have MDR-TB by culture increased the odds of having mixed MTB infection. The inefficiency of GeneXpert in detecting rifampicin resistance has been reported by several studies [40]. The major cause of false results could be due to the presence of rifampicin-resistant strains at low populations which is undetectable by GeneXpert [40]. This could be due to the presence of rifampicin mutations that occur outside the rifampicin resistance detectable regions [41]. In addition, this could also be due to the presence of disputed mutations which can only be expressed phenotypically but not Genotypically according to Torrea *et al* [42] and Eddabra & Neffa [43]. Hence the false negative resistance observed could be due to the presence of mixed MTB infection in the form of hetero-resistance.

Our study had some limitations considering that it used stored samples which may have impacted on the accuracy of the tests used, however, since these are DNA-based, we think that it is minimal to significantly affect our findings. The strength of our study is that we used sputum samples for determining mixed MTB infection. A previous study found that culture alters the mycobacterial population and underestimates the prevalence of mixed infection [44].

## CONCLUSION

The presence of mixed-MTB infection may affect the performance of the GeneXpert test but not for LPA. In determining resistance among MTB patients, rapid resistance-determining methods may complement each other. In case of inconclusive results among patients with high pre-test probability, an alternative rapid method, possible LPA should be considered.

## Supplementary Material

Supplement 1

## Figures and Tables

**Table 1:** Descriptive characteristics of participants and prevalence of Mixed MTB infection

Characteristics		% percentage N=53	Frequency (n)	Positive for Mixed TB infection n (%)	P-Value
**Sex**	Male	58.5	31	3 (9.7)	0.831
	Female	41.5	22	2 (9.1)	
**Age**	Young adults (18–30)	34.0	18	1 (5.5)	0.240
	Middle age adults (31–45)	56.6	30	4 (13.3)	
	Old age adults (>45)	9.4	5	0 (0.0)	
**MTB Lineage**	1	3.8	2	0 (0.0)	0.388
	2	7.5	4	1 (25.0)	
	3	26.4	14	1 (7.1)	
	4	62.3	33	3 (9.1)	

Effect of Mixed MTB infection on the performance of Rapid molecular diagnostic tests

**Table 2:** Comparison of Agar Proportional Method results with Line probe assay and GeneXpert test results

		Agar Proportional Method (APM)					
		MDR	RR	IR	S (I+R)	Indet	Total
		30					
LPA	MDR	25	23				
	RR		19	0			
	IR			4	0		
	S (I+R)				3	0	
	Indet					1	53
	Total						53
	Test Affected			4	3	1	7

GeneXpert			53				
	RR		48		0		
	S				3	0	
	Indet					2	53
	Total						53
	Test Affected				3	2	5

**MDR** – Multidrug Resistant, **RR** – Rifampicin Resistant, **IR** – Isoniazid Resistant, **I** – Isoniazid, **R** – Rifampicin, **S** – Sensitive, **Indet** – Indeterminate, **LPA**- Line Probe Assay

**Table 3:** Bivariate analysis of LPA and XPT results affected due to mixed MTB infection

	Variable		% participants N=53	Frequency (n)	LPA/XPT results Affected n (%)	unadjusted OR (95% CI)	P-Values
LPA	Mixed TB infection	No	90.6	48	5 (10.4)	1.071	0.954
		Yes	9.4	5	2 (40.0)	(0.103–11.130)	
XPT	Mixed TB infection	No	90.6	48	2 (4.2)	7.556	0.039*
		Yes	9.4	5	3 (60.0)	(0.886–64.445)	

LPA= Line Probe Assay, XPT= GeneXpert, MTB = *M. tuberculosis,* OR= Odds Ratio, CI= Confidence Interval

**Table 4:** Factors associated with mixed MTB infection among RR-TB patients

Variable		Participants (n/%) N=53	Frequency (n)	Positive for Mixed TB infection n (%)	Unadjusted* OR (95% CI)	P-Values
**MDR**	No	54.8	23	2 (8.7)	1.071 (0.103–11.130)	0.954
	Yes	45.2	30	3 (10.0)		
**XPT test results**	RR D	92.9	48	3 (6.3)	24.00 (1.66–347.89)	0.033
	RR ND	7.1	5	2 (40.0)		
**HIV**	Negative	61.9	37	1 (2.7)	8.333 (0.84–82.86)	0.040
	Positive	38.1	16	4 (25.0)		

RR – Rifampicin Resistance ND – Not Detected D – Detected XPT – Xpert MTB/RIF, MDR –Multi-Drug Resistant, MTB = *M. tuberculosis*, OR= Odds Ratio, CI= Confidence Interval
