# Supplementary material for: Effect of mixed Mycobacterium tuberculosis infection on rapid molecular diagnostics among patients starting MDR-TB treatment in Uganda
Source: Res Sq. 2023 Sep 28:rs.3.rs-3324330. Preprint. [Version 1] doi: 10.21203/rs.3.rs-3324330/v1 (PMC10571598; doi:10.21203/rs.3.rs-3324330/v1)
Supplement: Supplement 1 [file NIHPPrs3324330v1-supplement-1.pdf]

Supplementary Files

This is a list of supplementary files associated with this preprint. Click to download.

- SupportingInformation1.xlsx
